# Pathology and Pathogenesis of Metabolic Dysfunction-Associated Steatotic Liver Disease-Associated Hepatic Tumors

**DOI:** 10.3390/biomedicines11102761

**Published:** 2023-10-12

**Authors:** Yoshihisa Takahashi, Erdenetsogt Dungubat, Hiroyuki Kusano, Toshio Fukusato

**Affiliations:** 1 Department of Pathology, School of Medicine, International University of Health and Welfare, Narita 286-8686, Japan; dungubat.erdenetsogt.3p@tokyo-med.ac.jp (E.D.); h-kusano@iuhw.ac.jp (H.K.); 2 Department of Pathology, School of Biomedicine, Mongolian National University of Medical Sciences, Ulaanbaatar 14210, Mongolia; 3 General Medical Education and Research Center, Teikyo University, Tokyo 173-8605, Japan; fukusato@med.teikyo-u.ac.jp

**Keywords:** nonalcoholic fatty liver disease, nonalcoholic steatohepatitis, metabolic dysfunction-associated steatotic liver disease, metabolic dysfunction-associated steatohepatitis, hepatocellular carcinoma, steatohepatitic hepatocellular carcinoma, hepatocellular adenoma, intrahepatic cholangiocarcinoma

## Abstract

Nonalcoholic fatty liver disease (NAFLD) is characterized by excessive fat accumulation in the livers of patients without a history of alcohol abuse. It is classified as either simple steatosis (nonalcoholic fatty liver) or nonalcoholic steatohepatitis (NASH), which can progress to liver cirrhosis and hepatocellular carcinoma (HCC). Recently, it was suggested that the terms “metabolic dysfunction-associated steatotic liver disease (MASLD)” and “metabolic dysfunction-associated steatohepatitis (MASH)” should replace the terms “nonalcoholic fatty liver disease (NAFLD)” and “nonalcoholic steatohepatitis (NASH)”, respectively, with small changes in the definitions. MASLD, a hepatic manifestation of metabolic syndrome, is rapidly increasing in incidence globally, and is becoming an increasingly important cause of HCC. Steatohepatitic HCC, a histological variant of HCC, is characterized by its morphological features resembling non-neoplastic steatohepatitis and is closely associated with underlying steatohepatitis and metabolic syndrome. Variations in genes including patatin-like phospholipase domain-containing protein 3 (PNPLA3), transmembrane 6 superfamily 2 (TM6SF2), and membrane-bound O-acyltransferase domain-containing protein 7 (MBOAT7) are associated with the natural history of MASLD, including HCC development. The mechanisms of HCC development in MASLD have not been fully elucidated; however, various factors, including lipotoxicity, inflammation, reactive oxygen species, insulin resistance, and alterations in the gut bacterial flora, are important in the pathogenesis of MASLD-associated HCC. Obesity and MASLD are also recognized as risk factors for hepatocellular adenomas, and recent meta-analyses have shown an association between MASLD and intrahepatic cholangiocarcinoma. In this review, we outline the pathology and pathogenesis of MASLD-associated liver tumors.

## 1. Introduction

Nonalcoholic fatty liver disease (NAFLD) is characterized by an excessive fat accumulation in the livers of patients without a history of alcohol abuse. NAFLD is classified as either simple steatosis (nonalcoholic fatty liver) or nonalcoholic steatohepatitis (NASH). The former is characterized by hepatic steatosis, while the latter is characterized by steatosis, inflammation, and hepatic cell injury. Recent online surveys and hybrid meetings, in which a total of 236 panelists from 56 countries participated, suggested that the terms “metabolic dysfunction-associated steatotic liver disease (MASLD)” and “metabolic dysfunction-associated steatohepatitis (MASH)” should replace the terms “nonalcoholic fatty liver disease (NAFLD)” and “nonalcoholic steatohepatitis (NASH)”, respectively. At the same time, the definition was changed, and the presence of at least one of five cardiometabolic risk factors became required for the diagnosis of MASLD [1]. In this paper, we use the terms NAFLD and NASH when we describe the data that are based on the diagnostic criteria of NAFLD and NASH. Otherwise, we use the terms MASLD and MASH. MASH can progress to liver cirrhosis and hepatocellular carcinoma (HCC) [2]. MASLD is the hepatic manifestation of metabolic syndrome [3]. The prevalence of MASLD is rapidly increasing worldwide in line with the increased prevalence of obesity. The global prevalence of NAFLD is estimated to be as high as 30% [4] and approximately 1.5–6% of the general population have NASH [5]. Recently, Allen et al. [6] followed 5123 patients with NAFLD (median age: 52 years, percentage of women: 53%) for a median of 6.4 years (range: 1–23 years) and found that 3% of patients with NAFLD progressed to cirrhosis in 15 years. Five hundred and seventy five (11.2%) patients died within the follow-up period, and 6% of deaths were liver-related.

According to statistics from 2020, liver cancer is the sixth most commonly diagnosed cancer and the third most common cause of cancer-related death globally [7]. HCC and intrahepatic cholangiocarcinoma (iCCA) account for approximately 80% and 10–15% of primary liver cancers, respectively [7,8,9]. Although hepatitis B virus (HBV) and hepatitis C virus (HCV) infections, alcohol abuse, aflatoxin, and schistosomiasis are the most common causes of HCC, MASLD is becoming an increasingly important cause of HCC [10,11,12,13,14].

Steatohepatitic HCC (SH-HCC) is a histological variant of HCC with morphological features resembling non-neoplastic steatohepatitis such as steatosis, inflammation, fibrosis, the ballooning of malignant hepatocytes, and Mallory–Denk bodies (eosinophilic irregular aggregates found in the cytoplasm of hepatocytes). It was first proposed in 2010, and its association with underlying steatohepatitis and metabolic syndrome has been pointed out, suggesting that there are characteristic histopathological features in MASLD-associated HCC [15,16].

HCC has a poor prognosis, and early diagnosis is important to ensure early intervention with minimal complications arising from its development. To achieve early diagnosis of MASLD-associated HCC, it is important to appropriately stratify patients with MASLD who are likely to develop HCC and perform elaborate surveillance. Clinical information, including disease activity and the stage, as well as genetic information will become important for the stratification of patients with MASLD. Furthermore, to develop new preventive and therapeutic strategies for MASLD-associated HCC, the elucidation of its pathogenesis is essential. Regarding the genetic predisposition to MASLD-associated HCC, variations in several genes, including *patatin-like phospholipase domain-containing protein 3* (*PNPLA3*), have been reported to be associated with the natural history of MASLD, including HCC development [17]. The mechanisms of HCC development in MASLD have not been fully elucidated; however, various factors, including inflammation, insulin resistance, and alterations in the gut bacterial flora, are considered important [18].

Hepatocellular adenoma (HCA) is a benign tumor originating from hepatocytes, whereas iCCA is a malignant liver tumor with biliary differentiation. In addition to oral contraceptives and anabolic steroids, obesity and MASLD have been recognized as risk factors for HCA [19,20]. Moreover, recent meta-analyses have shown an association between MASLD and iCCA [21,22]. In this review, we outline the pathology and pathogenesis of MASLD-associated HCC, HCA, and iCCA.

## 2. Epidemiology and Clinical Features of MASLD-Associated HCC

Younossi et al. [23] examined surveillance, epidemiology, and end results (SEER) registries (2004–2009) using Medicare-linkage files for HCC in the United States. In the study, 4929 HCC cases and 14,937 control cases were examined, of which 14.1% of the HCC cases were related to NAFLD. NAFLD was the third most common underlying condition after HCV infection and alcoholic liver disease in patients with HCC. Across the 6-year period (2004–2009), there was a 9% annual increase in the number of NAFLD-associated HCC cases. In addition, patients with NAFLD-associated HCC were older, had shorter survival times, had more frequent heart disease, and were more likely to die from primary liver cancer. Another study showed that NASH was the most rapidly growing indication for liver transplantation in patients with HCC in the United States [24]. Many cases of HCC that develop from cryptogenic cirrhosis are considered to be associated with MASLD [25].

The risk of HCC in patients with MASLD depends on the severity of MASLD. In a retrospective cohort study of 6508 patients with NAFLD diagnosed using ultrasonography in Japan, the cumulative rates of NAFLD-associated HCC were 0.02%, 0.19%, and 0.51% at 4, 8, and 12 years, respectively. The annual rate of new HCC cases was 0.043%. Overall, 184 patients were considered to have significant fibrosis (equivalent to stage 3–4), and the cumulative rate of HCC was significantly higher in this group (hazard ratio (HR): 25.03, 95% confidence interval (CI): 9.02–69.52) [26]. However, a significant number of patients with MASLD-associated HCC have no evidence of cirrhosis [27]. The annual cumulative incidence of HCC was 2.6% in patients with NASH-associated cirrhosis and 4.0% in patients with HCV-associated cirrhosis [28]. The five-year incidence of HCC has been reported to be 11.3% and 30.5% in NASH-associated and HCV-associated cirrhosis, respectively [29]. These data suggest that MASH-associated cirrhosis has a lower incidence of HCC than HCV-associated cirrhosis.

## 3. SH-HCC: A Histological Variant of HCC That Is Closely Associated with MASLD

### 3.1. Clinical Features

SH-HCC is a histological variant of HCC that is reported to be strongly associated with MASLD. In 2010, Salomao et al. [30] noted a distinctive histological variant of HCC with features resembling non-neoplastic steatohepatitis, including large droplet steatosis, the ballooning of malignant hepatocytes, Mallory–Denk bodies, inflammation, and pericellular fibrosis in explant livers with chronic hepatitis C, and they named it SH-HCC. This variant was found in 22 (35.5%) of 62 HCC cases. Fourteen (63.6%) of the 22 SH-HCC cases had at least one known risk factor for MASLD/MASH, including diabetes, obesity, hypertension, and hyperlipidemia, and in 14 (63.6%) of the 22 SH-HCC cases, the non-neoplastic liver showed MASLD/MASH changes superimposed on otherwise typical features of chronic hepatitis C. In a follow-up study, the same group examined all HCCs diagnosed based on resection and explant specimens over 3.5 years at their institution. The SH-HCC variant was present in 16 (13.5%) of 118 cases. All but one case of SH-HCC occurred in patients with underlying steatohepatitis. SH-HCC was diagnosed in 35.7% of patients with either NASH or alcoholic liver disease, and its frequency was only 1.3% in patients with other chronic liver diseases. The SH-HCC group had significantly more risk factors for metabolic syndrome (2.44 vs. 1.48, *p* = 0.01) and a higher percentage of patients with at least three metabolic syndrome components (50% vs. 22.5%, *p* = 0.02) than the conventional HCC group. Thus, SH-HCC was suggested to be strongly associated with underlying steatohepatitis and metabolic syndrome [31]. In a study by Jain et al. [32], SH-HCC was identified in 19 (18.8%) of 101 HCC cases. Most SH-HCC cases were associated with metabolic risk factors, such as obesity, diabetes, hypertension, and hyperlipidemia. Although an association between SH-HCC and MASLD and metabolic syndrome has been suggested in many studies, it is noteworthy that SH-HCC occasionally develops in patients without MASLD or metabolic syndrome. Yeh et al. [33] examined 12 SH-HCC cases that were not associated with metabolic syndrome and background steatosis and detected the loss of 9q12–31.1 in a subset of cases, a finding that had not been previously reported in HCC. Moreover, a recent study reported that the frequency of SH-HCC was almost similar between NASH-associated HCC and alcoholic steatohepatitis-associated HCC, suggesting that SH-HCC is not specific to NAFLD-associated HCC [16].

### 3.2. Pathological Features

**Figure 1:**
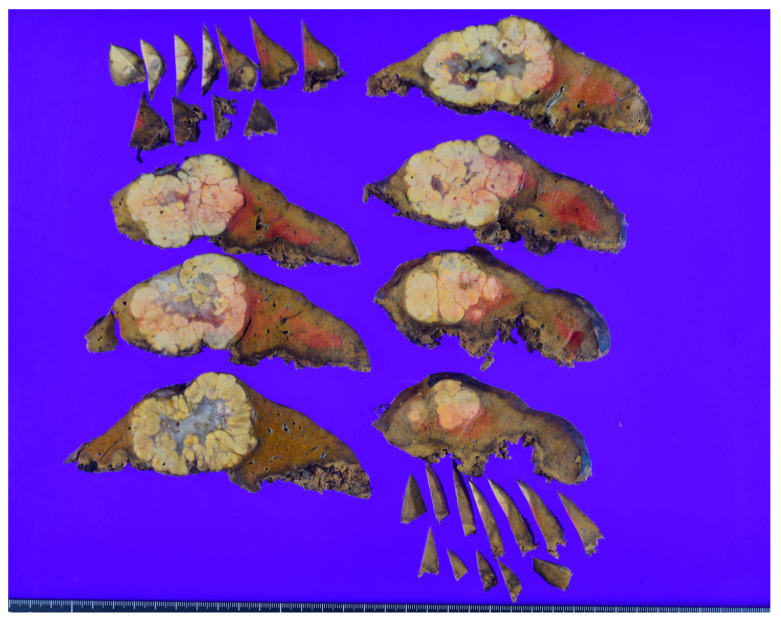
Macroscopic appearance of a case of steatohepatitic hepatocellular carcinoma (SH-HCC). A golden yellow-colored nodular tumor is observed in the liver (original photograph).

**Figure 2:**
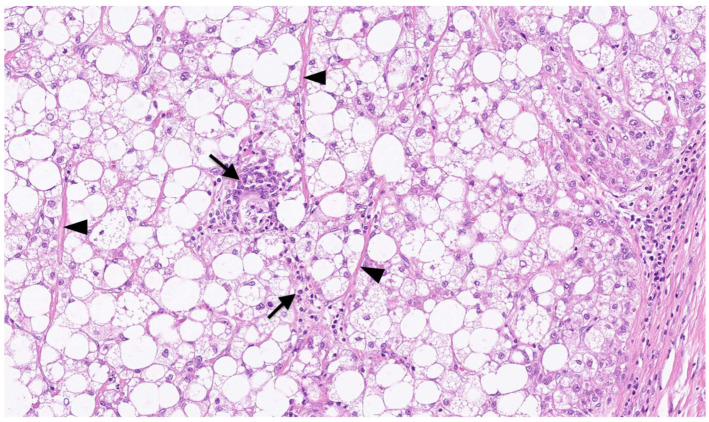
Microscopic appearance of SH-HCC. Large droplet steatosis, inflammation (arrows), and fibrosis (arrowheads) are observed in the tissue of hepatocellular carcinoma (HCC). This is an original photomicrograph with a magnification of ×125.

**Figure 3:**
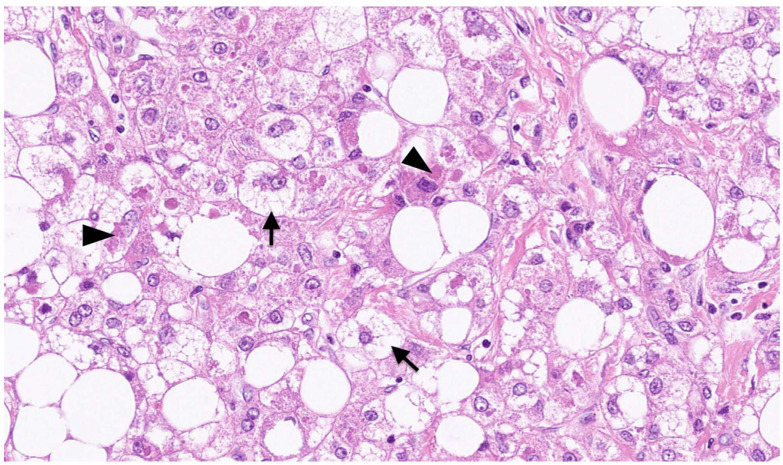
Microscopic appearance of SH-HCC. Ballooning of malignant hepatocytes (arrows) and Mallory–Denk bodies (arrowheads) are observed in the tissue of HCC. This is an original photomicrograph with a magnification of ×260.

On macroscopic examination, SH-HCC is golden-yellow in color, reflecting steatosis, and is slightly firmer than conventional HCC, reflecting fibrosis [31] (Figure 1). The tumors are nodular and well demarcated, and the size ranges from 0.5 to 11 cm [15]. Microscopically, SH-HCC is characterized by large-droplet steatosis, inflammation, fibrosis, ballooning, and Mallory–Denk bodies, which are characteristic features of steatohepatitis (Figure 2 and Figure 3). The pattern of fibrosis is trabecular (thick bundles of fibrous tissue within the tumor) or pericellular (thin strands of fibrosis with a “chicken-wire” appearance) [30]. Although the diagnostic criteria for SH-HCC have not been established, at least 5% or 50% of the tumor area must show steatohepatitic features [15,30,31]. SH-HCC displays a less aggressive histological phenotype, lacking satellite nodules and microvascular invasion [34].

### 3.3. Immunohistochemical Features

Few studies have examined the immunohistochemical features of SH-HCC. Glypican-3 was positive in 72.2–85.7% of SH-HCC cases, and the staining pattern was cytoplasmic or canalicular [30,32]. Salomao et al. [30] reported that the positive rates for glutamine synthetase (GS) and heat shock protein (HSP) 70 in SH-HCC were 100% and 57.1%, respectively. However, in a study using tissue microarrays by Ando et al. [35], the overexpression rate of GS and the incidence of nuclear accumulation of β-catenin in SH-HCC were only 4.3% and 5.7%, respectively, which were significantly lower than those in conventional HCC. In our study, serum amyloid A (SAA), which is characteristic of one subtype of HCA, was frequently overexpressed in SH-HCC [36]. SAA-positive HCCs frequently showed features of SH-HCC but less frequently showed nuclear β-catenin accumulation and GS immunopositivity (unpublished data).

### 3.4. Molecular Features

SH-HCC characteristically lacks Wnt/β-catenin pathway activation (lacks *CTNNB1* mutations and shows low GS expression). In contrast, the IL-6/JAK/STAT pathway is frequently activated with positive immunohistochemical staining for the C-reactive protein (CRP). Although the histological appearance of SH-HCC may suggest the dysregulation of metabolic processes, significant changes in the genes involved in fatty acid synthesis, glycolysis, or neoglucogenesis have not been observed [34].

### 3.5. Prognosis

The data on the prognosis of patients with SH-HCC are limited. However, no significant differences were observed in the overall survival, disease-free survival, development of metastatic disease, and local recurrence between SH-HCC and conventional HCC groups [31,37].

## 4. Pathological Characteristics of the Background Liver Tissue in MASLD-Associated HCC

In the pathological diagnosis of MASLD-associated HCC, it is noteworthy that steatosis and neuroinflammatory reactions in MASLD may disappear as fibrosis progresses (burn-out MASH) [38]. MASLD is considered a leading cause of cryptogenic cirrhosis [38], and a link between HCC in cryptogenic cirrhosis and MASLD has been suggested [39]. As mentioned previously, MASLD-associated HCC may develop in non-cirrhotic livers. A lower prevalence of cirrhosis has been reported for NAFLD-associated HCC (58.3–77.2%) compared with HCCs associated with other etiologies including alcoholic liver disease and chronic hepatitis C [27,40,41]. Iron deposition in the liver is more frequent in patients with NASH-associated cirrhosis and HCC than in the HCC-free controls, suggesting that iron overload is associated with the development of HCC in NASH-associated cirrhosis [42].

## 5. Genetic Predisposition of MASLD and MASLD-Associated HCC

High-throughput technologies including next-generation sequencing and DNA microarrays have advanced, and they are useful for detecting genetic variants and single-nucleotide polymorphisms (SNPs) [43,44]. Variations in various genes, such as *PNPLA3*, *transmembrane 6 superfamily 2* (*TM6SF2*), *membrane-bound O-acyltransferase domain-containing protein 7* (*MBOAT7*), and *hydroxysteroid 17-beta dehydrogenase 13* (*HSD17B13*) are associated with the natural history of MASLD [17]. *PNPLA3* encodes a triacylglycerol lipase that mediates triacylglycerol hydrolysis in the adipocytes. The *PNPLA3* rs738409C>G polymorphism (I148M) is associated with the histological severity (steatosis, portal and lobular inflammation, Mallory–Denk bodies, NAFLD activity score, and fibrosis) of NAFLD [45,46,47]. This genetic variant is also associated with NAFLD-associated HCC, and GG homozygotes exhibit a five-fold increased risk of HCC compared with CC homozygotes [48].

The precise function of TM6SF2 remains unclear. Dongiovanni et al. [49] reported that the carriers of the *TM6SF2* rs58542926 C>T polymorphism (E167K) had more severe steatosis, necroinflammation, ballooning, and fibrosis in the liver and were more likely to have NASH (odds ratio (OR): 1.84, 95% CI: 1.23–2.79) and advanced fibrosis (OR: 2.08, 95% CI: 1.20–3.55) compared with non-carriers after the adjustment for confounders, although they had a lower incidence of cardiovascular disease. The association of the genetic variant with hepatic steatosis, NASH, and hepatic fibrosis has been confirmed in other studies [50,51,52]. The expression of the TM6SF2 protein is markedly decreased in the livers of patients with NAFLD compared to that in normal people, and TM6SF2 immunoreactivity is decreased in people with at least one copy of the T allele [52]. A recent meta-analysis suggested a significant association between *TM6SF2* rs58542926 T/C polymorphism and HCC [53]. The genetic variant of *TM6SF2* promotes the expression of the inflammatory cytokines interleukin (IL)-2 and IL-6 [54] and affects the cell cycle of HCC tumor cells [55]. The association between the *TM6SF2* genetic variant and MASH and HCC may be mediated by these mechanisms.

*MBOAT7* encodes a protein involved in the re-acylation of phospholipids as part of the phospholipid remodeling pathway and is located near the *transmembrane channel like 4* (*TMC4*) gene. Mancina et al. [56] reported that the *MBOAT7-TMC4* rs641738 C>T variant was associated with increased hepatic fat content, more severe liver damage, and fibrosis. MBOAT7, but not TMC4, was highly expressed in the liver, and the *MBOAT7* rs641738 T allele was associated with lower protein expression in the liver. In another study, the rs641738 T allele was associated with NAFLD-associated HCC (OR: 1.65, 95% CI: 1.08–2.55), particularly in those without advanced fibrosis [57]. However, in one study, there was no evidence of an association between rs641738 and NAFLD or disease severity [58]. Further studies are required to elucidate the association between the genetic variant and MASLD.

*HSD17B13* encodes a liver-specific lipid droplet-associated protein. Abul-Husn et al. [59] reported that the *HSD17B13* rs72613567 T>TA variant was associated with a reduced risk of alcoholic liver disease and alcoholic and nonalcoholic cirrhosis. This variant was also associated with a reduced risk of NASH, but not steatosis. In a subsequent study, the protective effect of this variant was confirmed for alcoholic liver disease, NAFLD, and hepatitis C. In patients with alcoholic liver disease, the proportion of TA allele carriers with HCC was significantly lower than that in patients with chronic liver disease without HCC, even after adjusting for confounders [60].

Other genetic variants have also been reported to be associated with MASLD/MASH and MASLD-associated HCC. Eldafashi et al. [61] reported that *programmed cell death protein 1* (*PDCD1*) SNPs (rs7421861 and rs10204525) were specifically associated with NAFLD-HCC risk, regardless of cirrhosis, although *PNPLA3* and *TM6SF2* SNPs were associated with both the progression to cirrhosis and NAFLD-HCC development. Meroni et al. [62] evaluated the effect of the rs599839 A>G variant in the *cadherin EGF LAG seven-pass G-type receptor 2* (*CELSR2*)—*proline/serine-rich coiled-coil protein 1* (*PSRC1*)—*sortilin 1* (*SORT1*) gene cluster in 1426 NAFLD patients, of whom 131 had HCC. As a result, the minor G allele was associated with a higher risk of HCC, independent of the fibrosis severity (OR: 5.62, 95% CI: 1.77–17.84), poor prognosis, and advanced tumor stage. Dongiovanni et al. [63] reported that the *ectonucleotide pyrophosphatase/phosphodiesterase 1* (*ENPP1*) K121Q and *insulin receptor substrate 1* (*IRS-1*) Q972R polymorphisms predisposed patients with NAFLD to liver damage and decreased hepatic insulin signaling. Additionally, Musso et al. [64] reported that the 45TT and 276GT/TT genotypes of the adiponectin gene were more prevalent in patients with NAFLD than in the controls and independently predicted the severity of liver disease in NASH. These genotypes exhibited a blunted postprandial adiponectin response.

## 6. Mechanisms of HCC Development in MASLD

Various factors, including lipotoxicity, the activation of systemic and local inflammatory and immune pathways, reactive oxygen species, insulin resistance, and the alterations in the gut bacterial flora, are associated with the pathogenesis of MASLD-associated HCC [18]. The accumulation of fat in hepatocytes causes chronic inflammation and the generation of reactive oxygen species in the liver via lipotoxicity, which may induce HCC via DNA mutations. Furthermore, MASLD, a hepatic manifestation of metabolic syndrome, is strongly associated with systemic insulin resistance, and compensatory hyperinsulinemia and insulin signaling may induce HCC via various carcinogenic pathways [65]. Gut microbiota are attracting attention as a therapeutic target of MASLD via probiotics. In addition, the pharmacological effects of fibroblast growth factor (FGF) 21 on obesity-associated diseases are also becoming a research focus. The application of microRNAs (miRNAs) in MASLD therapy as oligonucleotide therapeutics is expected. Epigenetics is also a research hotspot due to its association with carcinogenesis and as an application to cancer therapy. Therefore, we summarize their roles in HCC development in MASLD below.

Changes in gut microbiota are important pathogenic mechanisms in MASLD-associated HCC. Ponziani et al. [66] examined the gut microbiota profiles of a consecutive series of patients with NAFLD-associated cirrhosis and HCC, patients with NAFLD-associated cirrhosis without HCC, and in the healthy controls. Bacteroides and Ruminococcaceae increased, whereas Bifidobacterium decreased in the HCC group. Patients with HCC had increased levels of fecal calprotectin, a marker of intestinal inflammation. Elevated serum levels of lipopolysaccharides (LPS) derived from Gram-negative bacterial surfaces have been demonstrated in NAFLD [67]. LPS acts via the toll-like receptor (TLR) 4 and promotes HCC by mediating increased proliferation, the expression of the hepatomitogen epiregulin, and the prevention of apoptosis [68]. Deoxycholic acid (DCA), a gut bacterial metabolite, is a DNA-damaging agent that plays an important role in the development of obesity-associated HCC. Dietary or genetic obesity increases DCA levels by altering the gut microbiota. The enterohepatic circulation of DCA induces a senescence-associated secretory phenotype in hepatic stellate cells, which in turn secretes various inflammatory and tumor-promoting factors in the liver and facilitates HCC development in mice exposed to chemical carcinogens [69]. Behary et al. [70] showed that the extracts from the microbiota of patients with NAFLD-associated HCC, but not the control groups, elicited a T cell immunosuppressive phenotype, characterized by the expansion of regulatory T cells and the attenuation of CD8+ T cells, suggesting the modulation of the peripheral immune response.

FGF21 is known to increase the energy expenditure, fat utilization, and lipid excretion, causing weight loss, increased insulin sensitivity, decreased blood glucose and lipid levels, and the amelioration of hepatic steatosis [71,72,73,74]. We recently showed that the hepatic expression levels of *FGF21* are higher in female TSOD and db/db mice (animal models of MASLD) than in their male counterparts, suggesting that FGF21 levels may be one of the causes of the male predominance of MASLD in reproductive age [75]. The deficiency of FGF21 promotes obesogenic diet-induced HCC in mice [76]. Furthermore, it has recently been shown that the lack of FGF21 promotes the MASH-HCC transition via hepatocyte-TLR4-IL-17A signaling [77]. *Cell cycle-related kinase* (*CCRK*), an androgen receptor-driven oncogene, collaborates with obesity-induced proinflammatory signaling and promotes MASLD-associated hepatocarcinogenesis [78].

miRNAs are also associated with the development of MASLD-associated HCC. Guo et al. [79] compared the clinical characteristics and unbiased expression profiles of 233 miRNAs in 36 liver biopsy specimens stratified by the disease severity of NAFLD. The expression levels of miR-301a-3p and miR-34a-5p increased and those of miR-375 decreased with the disease progression. Increased miR-301a and decreased miR-375 expression was also observed in 134 HCC samples in The Cancer Genome Atlas, suggesting that the miRNA expression pattern is associated with HCC development. In a subsequent animal study, hepatocyte miR-34a was shown to regulate the development and progression of MASLD by inducing lipid absorption, lipogenesis, inflammation, and apoptosis but inhibiting fatty acid oxidation [80]. miR-21 is one of the most frequently upregulated miRNAs in liver diseases, such as MASLD and HCC, and it plays multiple oncometabolic roles in MASLD-associated HCC via PI3K/AKT, TGF-β, and STAT3 signaling [81]. miR-122 accounts for 70% of the total miRNAs in the liver, and mice lacking the gene encoding miR-122a are viable but develop temporally controlled steatohepatitis, fibrosis, and HCC. The male-to-female ratio of HCC incidence in the mice was 3.9:1, reflecting the disease incidence in humans [82]. miR-223 has anti-inflammatory effects, and the genetic deletion of miR-223 induces a full spectrum of MASLD, including steatosis, inflammation, fibrosis, and HCC, in long-term high-fat diet-fed mice [83]. miR-22 has complicated effects on liver diseases; it inhibits the expression of FGF21 and its receptor, and hepatic miR-22 overexpression enhances diet- and alcohol-induced steatosis [84,85]. Simultaneously, miR-22 acts as a liver cancer suppressor [85]. The detailed effects of miR-22 on MASLD-associated HCC need to be elucidated in future studies. miRNAs are promising diagnostic and prognostic biomarkers that are useful in staging various hepatic disorders that could lead to liver cancer. In particular, circulatory miR-147b, miR-221, miR-512-5p, miR-542, miR-552-3p, miR-650, and miR-676-3p expression profiles are useful biomarkers for the diagnosis and staging of HCV-associated fibrosis, cirrhosis, and HCC [86,87].

The role of epigenetic changes in the development of MASLD-associated HCC is noteworthy. Li et al. [88] performed genetic and epigenetic data mining and system identification using the next-generation sequencing data and the corresponding DNA methylation profiles of liver cells in normal individuals and in patients with NAFLD and NASH, primary biliary cholangitis (PBC) and primary sclerosing cholangitis (PSC), and HCC, where they identified the genome-wide real genetic and epigenetic networks. They found that hepatocarcinogenesis via NAFLD and NASH was induced via DNA methylation of *histone H2B type 2-E* (*HIST2H2BE*), *heat shock protein family B (small) member 1* (*HSPB1*), *ribosomal protein L30* (*RPL30*), and *aldolase B* (*ALDOB*), as well as the regulation of miR-21 and miR-122.

*Cytotoxic T-lymphocyte-associated protein 4 (CTLA4), Ras association domain-containing protein 1 isoform A (RASSF1A), and signal transducer and activator of transcription 4 (STAT4)* genes regulate the cell cycle, apoptosis, and the autoimmune response against cancer [89]. Ali et al. [89] reported that the rs2073498 variation in the *RASSF1A* gene and the rs7574865 variation in the *STAT4* gene could make patients susceptible to HCV-associated HCC. Akt and topoisomerase-IIB could be therapeutic targets for various cancers including liver cancer [90,91].

## 7. MASLD-Associated HCA and iCCA

### 7.1. MASLD-Associated HCA

HCA is a benign tumor originating from hepatocytes and is classified as *HNF1A*-mutated HCA (H-HCA), inflammatory HCA (IHCA), β-catenin-mutated HCA (b-HCA), b-IHCA, sonic hedgehog HCA (shHCA), and unclassified HCA (UHCA) based on its genotypic and phenotypic features [92]. Although the representative risk factors for HCA are oral contraceptives and anabolic steroids, obesity and metabolic syndrome are also known risk factors for HCA and are especially associated with IHCA and shHCA [19,92,93,94,95]. HCA in obese patients often regresses after weight loss, especially after bariatric surgery, which may prevent the need for liver resection [96,97,98,99]. This confirms the importance of obesity in the development of HCA and suggests that weight loss may become the first therapeutic option for HCA in obese patients.

An association between MASLD/MASH and HCA, especially multiple HCA (adenomatosis), has been suggested [100,101,102,103]. Furlan et al. [104] reported that hepatic steatosis was observed in 14 (58%) of 24 patients who had hepatic adenoma compared with 7 (29%) of 24 patients who had hepatic hemangioma (*p* = 0.042). The frequency of steatosis was higher in patients with multiple hepatic adenomas (9/11, 82%) than in those with a single hepatic adenoma (5/13, 38%) (*p* = 0.047). However, it is uncertain whether MASLD/MASH is directly involved in the development of HCA or whether obesity and metabolic syndrome induce both MASLD/MASH and HCA.

As HCC may have steatohepatitic features (SH-HCC), HCA may have steatohepatitic features (SH-HCA). Liu et al. [105] examined the clinicopathological characteristics of patients with SH-HCA and found that 6 (14.6%) of the 41 HCA cases showed steatohepatitic morphology, of which three were H-HCA and three were IHCA. Compared with patients with conventional HCA, those with SH-HCA had a higher frequency of type 2 diabetes, obesity, and hypertension. Among the six SH-HCA cases, background liver tissue showed steatosis in three cases (50%) and steatohepatitis in one case (16.7%). In our study, SAA, which is characteristic of IHCA, was frequently overexpressed in SH-HCC, indicating a relationship between IHCA and SH-HCC [36].

### 7.2. MASLD-Associated iCCA

Cholangiocarcinoma (CCA) is classified into intrahepatic CCA (iCCA) and extrahepatic CCA (eCCA). Further, iCCA is classified into small- and large-duct types. The patterns of genomic alterations and potential oncogenic drivers differ between iCCA and eCCA and between small-duct- and large-duct-type iCCA [106]. Although PSC, liver flukes, and viral hepatitis are well-known risk factors for iCCA, an association between MASLD/MASH and iCCA has also been suggested [107,108]. In a meta-analysis by Wongjarupong et al. [21], NAFLD was associated with both iCCA (OR: 2.22, 95% CI: 1.52–3.24) and eCCA (OR: 1.55, 95% CI: 1.03–2.33). In a meta-analysis by Corrao et al. [22], NAFLD showed a significant association with iCCA (OR: 2.19, 95% CI: 1.48–3.25), but it did not show a significant effect on eCCA (OR: 1.48, 95% CI: 0.93–2.36). Although further examination of the association between MASLD and eCCA is necessary, MASLD appears to be a definite risk factor for iCCA. A recent study confirmed that MASLD exacerbates cholangitis and promotes iCCA in mice [109]. iCCA is characterized by a highly reactive desmoplastic stroma with a complex mechanism underlying the mutual interactions between tumor cells and the stromal compartment [110]. Although the pathogenic mechanisms of MASLD-associated iCCA have not been elucidated, iCCA development in patients with metabolic syndrome is characterized by osteopontin overexpression in the tumor stroma [111].

## 8. Conclusions and Future Perspectives

MASLD is increasing globally, in line with the increased prevalence of obesity; thus, the importance of MASLD as a cause of HCC is increasing. One of the characteristics of MASLD-associated HCC is that the frequency of cirrhosis in the background liver tissue is lower than that in HCC caused by other etiologies. This makes the surveillance of MASLD-associated HCC difficult. NAFLD-associated HCC is more often detected at a later stage and has a significantly shorter survival time than HCV-associated HCC [112]. Therefore, establishing a surveillance method for detecting HCC in patients with MASLD is an urgent task.

SH-HCC has been reported as a histological variant of HCC that is characteristic of MASLD-associated HCC. However, not all MASLD-associated HCCs are SH-HCCs, and HCCs of other etiologies, including alcoholic liver disease, may show morphological features of SH-HCC. Further investigations are necessary to elucidate the pathogenesis of SH-HCC. In particular, whether systemic abnormalities in lipid metabolism induce steatohepatitic changes in both non-tumorous and tumorous hepatic tissues, or whether special genetic alterations in the tumorous tissue induce steatohepatitic changes in HCC, should be elucidated. In the era of genomic medicine, further elucidation of genetic abnormalities characteristic of MASLD-associated HCC may enable the development of novel molecularly targeted therapies.

It has become clear that MASLD is associated not only with HCC but also with HCA and iCCA. However, the histopathological features and molecular abnormalities characteristic of MASLD-associated HCA have not yet been elucidated. Studies involving a large number of HCA cases with steatohepatitic morphology (SH-HCA) are warranted. Recent meta-analyses confirmed that NAFLD is a risk factor for iCCA. However, the histopathological features and molecular abnormalities characteristic of MASLD-associated iCCA have not yet been elucidated. As mentioned previously, iCCA is classified into small- and large-duct types with different histological and molecular features. iCCA can also be classified into inflammation and proliferation subclasses based on its molecular features, and this classification has a clinicopathological correlation [113]. Future studies should clarify which type and subclass of iCCA is associated with MASLD.
